# Supplementary material for: Ancient origins of arthropod moulting pathway components
Source: eLife. 2019 Jul 3;8:e46113. doi: 10.7554/eLife.46113 (PMC6660194; doi:10.7554/eLife.46113)
Supplement: Supplementary file 1. — Superphylum and/or phylum of the investigated species and the online repositories for each of the databases are also listed. [file elife-46113-supp1.docx]

| Species | Superphylum/  phylum | Biological sequence data repositories |
| --- | --- | --- |
| *Australostichopus mollis* | Deuterostomia/  Echinodermata | <http://ryanlab.whitney.ufl.edu/genomes/Amol/> |
| *Branchiostoma belcheri* | Deuterostomia/  Cephalochordata | <http://genome.bucm.edu.cn/lancelet/download_data.php> |
| *Branchiostoma floridae* | Deuterostomia/  Cephalochordata | [http://genome](http://genome/).jgi.doe.gov/Brafl1/Brafl1.download.html |
| *Ciona intestinalis* | Deuterostomia/  Chordata | http://genome.jgi.doe.gov/Cioin2/Cioin2.download.ftp.html |
| *Danio rerio* | Deuterostomia/  Chordata | http://mar2015.archive.ensembl.org/Danio_rerio/Info/Index |
| *Homo sapiens* | Deuterostomia/  Chordata | http://grch37.ensembl.org/Homo_sapiens/Info/Index |
| *Lytechinus variegatus* | Deuterostomia/  Echinodermata | http://www.echinobase.org/Echinobase/LvDownloads |
| *Mus musculus* | Deuterostomia/  Chordata | http://www.ensembl.org/Mus_musculus/Info/Index |
| *Patiriella regularis* | Deuterostomia/  Echinodermata | http://ryanlab.whitney.ufl.edu/genomes/Preg/ |
| *Ophionereis fasciata* | Deuterostomia/  Echinodermata | http://ryanlab.whitney.ufl.edu/genomes/Ofas/ |
| *Patiria miniata* | Deuterostomia/  Echinodermata | http://www.echinobase.org/Echinobase/PmDownload |
| *Ptychodera flava* | Deuterostomia/  Hemichordata | https://groups.oist.jp/molgenu/hemichordate-genomes |
| *Saccoglossus kowalevskii* | Deuterostomia/  Hemichordata | https://groups.oist.jp/molgenu/hemichordate-genomes |
| *Strongylocentrotus purpuratus* | Deuterostomia/  Echinodermata | http://www.echinobase.org/Echinobase/SpDownloads |
| *Caenorhabditis elegans* | Ecdysozoa/  Nematoda | http://www.ensembl.org/Caenorhabditis_elegans/Info/Index |
| *Drosophila melanogaster* | Ecdysozoa/  Arthropoda | http://www.ensembl.org/Drosophila_melanogaster/Info/Index |
| *Euperipatoides rowelli* | Ecdysozoa/  Onychophora | https://www.ncbi.nlm.nih.gov/Traces/wgs/?val=PXIH01#scaffolds |
| *Tribolium castaneum* | Ecdysozoa/  Arthropoda | http://metazoa.ensembl.org/Tribolium_castaneum/Info/Index |
| *Parhyale hawaiensis* | Ecdysozoa/  Arthropoda | https://figshare.com/articles/supplemental_data_for_Parhyale_hawaniensis_genome/3498104 |
| *Hypsibius dujardini* | Ecdysozoa/  Tardigrada | http://ensembl.tardigrades.org/Hypsibius_dujardini_nhd315/Info/Index |
| *Ramazzottius varieornatus* | Ecdysozoa/  Tardigrada | http://download.tardigrades.org/v1/sequence/ |
| *Parasteatoda tepidariorum* | Ecdysozoa/  Arthropoda | https://i5k.nal.usda.gov/content/data-downloads |
| *Peripatopsis capensis* | Ecdysozoa/  Onychophora | https://www.ncbi.nlm.nih.gov/Traces/wgs/?val=PXIH01#scaffolds |
| *Nematostella vectensis* | Cnidaria | http://metazoa.ensembl.org/Nematostella_vectensis/Info/Index |
| *Mnemiopsis leidyi* | Ctenophora | https://research.nhgri.nih.gov/mnemiopsis/ |
| *Trichoplax adhaerens* | Placozoa | http://metazoa.ensembl.org/Trichoplax_adhaerens/Info/Index |
| *Amphimedon queenslandica* | Porifera | http://amphimedon.qcloud.qcif.edu.au/ |
| *Isodiametra pulchra* | Xenacoelomorpha/Acoela | SRR2681926 |
| *Convolutriloba macropyga* | Xenacoelomorpha/Acoela | SRR2681679 |
| *Eumecynostomum macrobursalium* | Xenacoelomorpha/Acoela | SRR3105705 |
| *Diopisthoporus longitubus* | Xenacoelomorpha/Acoela | SRR3105704 |
| *Diopisthoporus gymnopharyngeus* | Xenacoelomorpha/Acoela | SRR3105703 |
| *Childia submaculatum* | Xenacoelomorpha/Acoela | SRR3105702 |
| *Monosiga brevicollis* | Choanoflagellata | https://genome.jgi.doe.gov/Monbr1/Monbr1.home.html |
| *Acanthochitona crinita* | Lophotrochozoa /Mollusca | SRS1859980 |
| *Antalis entalis* | Lophotrochozoa /Mollusca | SRS1859983/SRR330084 |
| *Barentsia gracilis* | Lophotrochozoa /Entoprocta | SRR1611554 |
| *Bathymodiolus platifrons* | Lophotrochozoa /Mollusca | <http://datadryad.org/resource/doi:10.5061/dryad.h9942/> |
| *Biomphalaria glabrata* | Lophotrochozoa /Mollusca | <https://www.vectorbase.org/organisms/biomphalaria-glabrata/> |
| *Bugula neritina* | Lophotrochozoa /Ectoprocta | SRR1237126/SRR1237127/SRR1237128 |
| *Capitella teleta* | Lophotrochozoa /Annelida | <ftp://ftp.ensemblgenomes.org/pub/metazoa/release-34/fasta/capitella_teleta/pep/> |
| *Chaetoderma sp* | Lophotrochozoa /Mollusca | SRR1505105 |
| *Chaetopleura apiculata* | Lophotrochozoa /Mollusca | SRR330421 |
| *Crassostrea gigas* | Lophotrochozoa /Mollusca | <ftp://ftp.ensemblgenomes.org/pub/metazoa/release-34/fasta/crassostrea_gigas/pep/> |
| *Diuronotus aspetos* | Lophotrochozoa /Gastrotricha | SRR2131262 |
| *Dreissena rostriformis* | Lophotrochozoa /Mollusca | Supplementary data herein |
| *Ennucula tenuis* | Lophotrochozoa /Mollusca | SRR331123 |
| *Entalina tetragona* | Lophotrochozoa /Mollusca | SRR2057018 |
| *Gadila tolmiei* | Lophotrochozoa /Mollusca | SRR331897 |
| *Graptacme eborea* | Lophotrochozoa /Mollusca | SRR2057020 |
| *Gymnomenia pellucida* | Lophotrochozoa /Mollusca | SRS1859979 |
| *Idiosepius notoides* | Lophotrochozoa /Mollusca | SRR5110534/SRR5110528 |
| *Kraussina rubra* | Lophotrochozoa /Brachiopoda | SRR2131392 |
| *Laevipilina hyalina* | Lophotrochozoa /Mollusca | SRR330425/SRR1505115 |
| *Leptochiton rugatus* | Lophotrochozoa /Mollusca | SRR1611558 |
| *Lineus longissimus* | Lophotrochozoa /Nemertea | SRR2682192 |
| *Lingula anatina* | Lophotrochozoa /Brachiopoda | SRR330440/ ftp://ftp.ensemblgenomes.org/pub/metazoa/release-34/fasta/lingula_anatina/pep/ |
| *Littorina littorea* | Lophotrochozoa /Mollusca | SRR331945 |
| *Loxosoma pectinaricola* | Lophotrochozoa /Entoprocta | SRR1611559 |
| *Lottia goshimai (Lottia cf. kogamogai)* | Lophotrochozoa /Mollusca | SRS1859984 |
| *Lottia gigantea* | Lophotrochozoa /Mollusca | <http://genome.jgi.doe.gov/Lotgi1/Lotgi1.download.ftp.html/> |
| *Membranipora membranacea* | Lophotrochozoa /Ectoprocta | SRR2131259 |
| *Mesodasys laticaudatus* | Lophotrochozoa /Gastrotricha | SRR1797883 |
| *Modiolus philippinarum* | Lophotrochozoa /Mollusca | <http://datadryad.org/resource/doi:10.5061/dryad.h9942/> |
| *Nautilus pompilius* | Lophotrochozoa /Mollusca | SRR330442 |
| *Neomenia megatrapezata* | Lophotrochozoa /Mollusca | SRR331899 |
| *Neomeniomorpha sp* | Lophotrochozoa /Mollusca | SRR331902 |
| *Nucula tumidula* | Lophotrochozoa /Mollusca | SRS1859987 |
| *Octopus bimaculoides* | Lophotrochozoa /Mollusca | <ftp://ftp.ensemblgenomes.org/pub/metazoa/release-34/fasta/octopus_bimaculoides/pep/> |
| *Octopus vulgaris* | Lophotrochozoa /Mollusca | SRR331946 |
| *Patinopecten yessoensis* | Lophotrochozoa /Mollusca | <http://mgb.ouc.edu.cn/novegene/html/download.php> |
| *Perotrochus lucaya* | Lophotrochozoa /Mollusca | SRR330462 |
| *Phoronis psammophila* | Lophotrochozoa /Phoronida | SRR1611565 |
| *Phoronis vancouverensis* | Lophotrochozoa /Phoronida | SRR1611566 |
| *Pinctada fucata* | Lophotrochozoa /Mollusca | <http://marinegenomics.oist.jp/pearl/viewer/download?project_id=20> |
| *Rhyssoplax olivacea (Chiton olivaceus)* | Lophotrochozoa /Mollusca | SRR618506 |
| *Rotaria socialis* | Lophotrochozoa /Rotifera | SRR2430028 |
| *Rotaria sordida* | Lophotrochozoa /Rotifera | SRR2430030 |
| *Rotaria tardigrada* | Lophotrochozoa /Rotifera | SRR2430032 |
| *Schistosoma mansoni* | Lophotrochozoa /Platyhelminthes | <ftp://ftp.sanger.ac.uk/pub/pathogens/Schistosoma/mansoni/genome/Gene_models/> |
| *Schmidtea mediterranea* | Lophotrochozoa /Platyhelminthes | [http://smedgd.stowers.org/downloads/#MAKER_annotations_8211_Protein_FASTA_files](http://smedgd.stowers.org/downloads/#MAKER_annotations_8211_Protein_FASTA_files) |
| *Scutopus ventrolineatus* | Lophotrochozoa /Mollusca | SRS1859986 |
| *Siphonaria pectinata* | Lophotrochozoa /Mollusca | SRR330463 |
| *Solemya velum* | Lophotrochozoa /Mollusca | SRR330465 |
| *Taenia solium* | Lophotrochozoa /Platyhelminthes | <http://parasite.wormbase.org/Taenia_solium_prjna170813/Info/Index/> |
| *Tubulanus polymorphus* | Lophotrochozoa /Nemertea | SRR1611583 |
| *Wirenia argentea* | Lophotrochozoa /Mollusca | SRS1859982 |
| *Yoldia limatula* | Lophotrochozoa /Mollusca | SRR330464 |
